# Identificação da velocidade anormal da dilatação de pupila como biomarcador de lesão cerebral em pacientes neurocríticos

**DOI:** 10.5935/0103-507X.20210065

**Published:** 2021

**Authors:** Prachi Singh, Sonia E. Stutzman, Aardhra Venkatachalam, DaiWai M. Olson, Arianna Barnes, Folefac D. Atem

**Affiliations:** 1 University of Texas at Southwestern Medical Center - Dallas, Texas, Estados Unidos.

**Keywords:** Neurociências/estatística & dados numéricos, Traumatismos do nervo óptico, Traumatismos do nervo oculomotor, Distúrbios pupilares, Manifestações neurológicas, Escala de coma de Glasgow

## Abstract

**Objetivo:**

Calcular as velocidades médias da dilatação de pupila para classificar a gravidade da lesão derivada da escala de coma de Glasgow, estratificada por variáveis de confusão.

**Métodos:**

Neste estudo, analisaram-se 68.813 exames das pupilas para determinar a velocidade normal de dilatação em 3.595 pacientes com lesão cerebral leve (13 - 15), moderada (9 - 12) ou grave (3 - 8), segundo a escala de coma de Glasgow. As variáveis idade, sexo, raça, tamanho da pupila, tempo de permanência na unidade de terapia intensiva, pressão intracraniana, uso de narcóticos, classificação pela escala de coma de Glasgow e diagnóstico foram consideradas confundidoras e controladas para análise estatística. Empregou-se regressão logística com base em algoritmo de classificação com aprendizado de máquina para identificar os pontos de corte da velocidade de dilatação para as categorias segundo a escala de coma de Glasgow.

**Resultados:**

As razões de chance e os intervalos de confiança desses fatores se mostraram estatisticamente significantes em sua influência sobre a velocidade de dilatação. A classificação com base na área sob a curva mostrou que, para o grau leve, na escala de coma de Glasgow, o limite da velocidade de dilatação foi de 1,2mm/s, com taxas de falsa probabilidade de 0,1602 e 0,1902 e áreas sob a curva de 0,8380 e 0,8080, respectivamente, para os olhos esquerdo e direito. Para grau moderado na escala de coma de Glasgow, a velocidade de dilatação foi de 1,1mm/s com taxas de falsa probabilidade de 0,1880 e 0,1940 e áreas sob a curva de 0,8120 e 0,8060, respectivamente, nos olhos esquerdo e direito. Mais ainda, para o grau grave na escala de coma de Glasgow, a velocidade de dilatação foi de 0,9mm/s, com taxas de falsa probabilidade de 0,1980 e 0,2060 e áreas sob a curva de 0,8020 e 0,7940, respectivamente, nos olhos esquerdo e direito. Esses valores foram diferentes dos métodos prévios de descrição subjetiva e das velocidades de dilatação previamente estimadas.

**Conclusão:**

Observaram-se velocidades mais lentas de dilatação pupilar em pacientes com escores mais baixos na escala de coma de Glasgow, indicando que diminuição da velocidade pode indicar grau mais grave de lesão neuronal.

## INTRODUCTION

The eyes are important organs because they allow us to process our world. They absorb light from the environment and convert it into signals, which then become images. Different amounts of light cause the pupils to constrict or dilate.^(1)^ The pupillary light reflex (PLR) is known as an objective marker of the amount of light input that the eye receives, and it is modulated by both the amount of input and the neurochemical perception of that light.^(2)^ Changes in the PLR may indicate neurological worsening or impending secondary brain injury. Changes in the ability of the pupil to constrict and dilate in response to light entering the eye may indicate a variety of disorders involving the afferent and efferent pathways (sympathetic stimulation, parasympathetic blockage, third cranial nerve-CNIII damage) or traumatic brain injuries (TBI).^(3, 4)^ Dilation velocity (DV) is one of several variables within the PLR, but it has not been well studied in acute neurological injury, primarily due to a lack of quantifying mechanisms.^(5)^

A deviation in DV may indicate a serious medical condition affecting the neurological system, such as mental health issues, strokes, infections, and neurodegenerative disorders.^(4, 6)^ This is especially crucial in the acute care setting, as changes occur quickly and patients require high-level monitoring. Associations of worsening medical condition have been studied with variables such as constriction velocity (CV) and the neurological pupillary index (NPi), but DV has not yet been evaluated thoroughly. These variables are all part of a concept called the PLR, and greater understanding of the specific variables is valuable

The PLR was difficult to evaluate in a standardized manner before the invention of a pupilometer. Previous PLR assessments were subjective and measured on a zero to four scale. Healthy individuals were described as having a 4+ response that was “brisk” and “large”.^(7)^ A common abbreviation used in healthcare is PERRL, or “pupils are equal, round, reactive to light”. Other words such as “unequal” and “sluggish” have also been used to describe pupillary responses.^(8)^ However, these terms are subjective descriptions and ill-defined.

Automated infrared pupillometry (AIP) offers the possibility to measure DV in millimeters per second (mm/s) and quantifies pupil recovery to normal size (dilation) after constriction.^(9, 10)^ The NeurOptics^®^ Pupillometer measures PLR metrics objectively with high reliability and gives values such as CV and DV.^(11, 12)^ To date, values to compare DV and neuronal injury have not been standardized in a large sample. Various conditions affect the brain and pupillary response (sensory, motor) in different ways, so DV may serve as an indicator of underlying complications.^(13)^ The Neurological Pupil index™ (NPi™), a measure combining different values of the PLR, has been used for diagnostic and prognostic assessments in recent years.^(14)^ An NPi score ≥ 3.0 was considered to be normal, while values < 3 were scored as abnormal. However, a study by Shoyombo et al.^(15)^ found that 17% of patients with a normal NPi had a clinical mismatch with abnormal pupillary reactions.

The Glasgow coma scale (GCS) has been used to evaluate neurologic impairment in patients with craniocerebral injury. Total GCS scores range from a low of three (worst) to a high of 15 (best) by evaluating 3 items: best verbal response (range one to five), best motor response (range one to six), and best eye-opening response (range one to four). Pupil reactivity and GCS showed a direct, negative relationship: as GCS decreased, pupil reactivity decreased and mortality worsened.^(16)^ With all of this in mind, this study attempts to determine the critical pupillary DV values in patients with a wide range of neurocritical illnesses and in three GCS classifications. This serves to better understand a poorly defined variable, DV, which can help to further specify neuronal injury. We are interested in estimating the risk score function p(x), where p(x) = P(D =1| X(t) = x) and 0 < p(x) < 1 is the disease probability, given X = x.

The purpose of this study was to employ common machine-learning techniques to quantify critical normal DVs based on GCS classification and their use as biomarkers of neuronal injury. In doing so, the critical DV value can be used as a cutoff for comprehending normal DV in patients with varying brain injuries.

## METHODS

This study was conducted as retrospective analysis of subject data obtained from the END Panic registry (NCT02804438), which is a prospective multicenter registry that collects AIP data from three large urban medical centers, as well as patients admitted to the neurocritical care units. The registry has been fully described in a prior publication.^(17)^ In brief, AIP readings provided PLR data parameters such as pupillary reflexes, DV, CV, pupillary latency, and NPi.^(18)^ This study served to determine benchmark values for pupillary DVs depending on GCS. The GCS stratified patients based on brain injury and was classified as mild (GCS 13 - 15), moderate (GCS 9 - 12), and severe (GCS 3 - 8). Patient age, sex, race, primary diagnosis requiring neurological hospitalization, narcotic use, presence and location of intracranial pressure (ICP), length of stay in the intensive care unit (ICU), and size of pupil were controlled for. Patients classified into GCS exhibit a gamut of DVs, and the critical value of DV calculated allows for differentiation between DVs above and below that score. It also shows an association of score range with specific GCS range, and it gives a good basis to start analyzing DV values and neurological insult.

### Statistical analysis

Descriptive analyses were performed on baseline characteristics and analyzed as follows: age as a continuous predictor; sex as a categorical predictor; race as a categorical predictor with four levels (Caucasians, African American, Asian, and Other); primary diagnosis as a categorical predictor with five levels (hemorrhagic stroke, TBI, tumor, ischemic stroke, and infection/LMN); narcotics as a binary predictor; ICP, ICU length of stay, and pupil size were all analyzed as continuous predictors. The logistic regression approach was validated to predict the presence of injury. Several threshold values were tested, and each value had a corresponding true positive rate (TPR), and a false positive rate (FPR) was performed on both the training and validation data. Thus, to improve precision, we present results from the entire data set. Receiver operative characteristic (ROC) curves were created to show the relationship between the FPR and sensitivity. Thus, for a d-dimensional vector of possible dependent covariates, the binary outcome (0,1) reflects the absence or presence of binary injury. We were interested in finding a threshold cutoff point for the score function, given the probability function of having a brain injury for the set of covariates. A perfect model that completely separates the disease outcomes would have a 100% TPR, and the area under the curve (AUC) would be equal to one. The calculated AUC determined the sweet spot for the DV value. The AUC values are interpreted as follows: AUC = 0.5 is noninformative; 0.5 < AUC < 0.7 is less accurate; 0.7 < AUC < 0.9 is moderately accurate; 0.9 < AUC < 1 is highly accurate; and 1 is perfect.^(19)^ Our goal was to determine the sweet spot with an AUC > 0.75.^(20)^ This was the cutoff decided upon due to the typical interpretation of AUCs. A line equal to 1 would indicate a perfect association, while a score of 0.5 would indicate no association. Thus, the value of 0.75 shows that there is a significantly positive association. Using this method, we can determine the normal DV depending on GCS. Right and left eye readings for every GCS level were evaluated separately. Statistical significance was defined as a p-value < 5% or 95% interval estimates excluding the null value as appropriate. Statistical analyses were performed using Statistical Analysis System (SAS) version 9.4 (SAS Institute, Cary, NC).

## RESULTS

The 3,595 subjects were primarily 51% female; 14% were African American, 3% Asian, 78% Caucasian, and 5% other; and 89% identified as Hispanic (Table 1). At presentation, the GCS scores were categorized as severe in 17% of patients, moderate in 12%, and mild in 71% of patients.

**Table 1:** Baseline characteristics

Variable	n (%)
Gender	
Female	1,838 (51)
Male	1,756 (49)
Ethnicity	
Hispanic	3,147 (89)
Nonhispanic	380 (11)
Race	
African American	474 (14)
Asian	113 (3)
Caucasian	2,698 (78)
Other	191 (5)
Diagnosis	
Hemorrhagic stroke	893 (25)
TBI	126 (4)
Tumor	936 (26)
Ischemic stroke	646 (18)
Infection/LMN	973 (27)
Admission injury score	
Mild (GCS 13 - 15)	2,544 (71)
Moderate (GCS 9 - 12)	439 (12)
Severe (GCS 3 - 8)	612 (17)

TBI - traumatic brain injury; LMN - lower motor neuron; GCS - Glasgow Coma Scale.

Parameter estimates of DV by injury severity, the eye evaluated, and confounders are detailed in tables 2A and 2B. For mild GCS, in the left eye, all variables except sex, being Caucasian, and a tumor diagnosis were statistically significant (p < 0.05). In the right eye, all variables except being Caucasian were statistically significant. For moderate GCS, in the left eye, all variables except ICP, ICU length of stay, and narcotics were statistically significant. On the right, all variables, except diagnosis with hemorrhagic stroke, narcotics, ICP, and ICU length of stay, were statistically significant.

**Table 2A:** Statistically significant parameter estimate based on acceptable threshold dilation velocity for the left pupil

	Adjusted model		
	OR	95%CI	p value
Left pupil, mild GCS			
Age	1,011	1,009 - 1,014	< 0,0001
Race			
4 *versus* 1	0.532	0.442 - 0.639	< 0,0001
2 *versus* 1	0.555	0.494 - 0.624	< 0,0001
Primary diagnosis			
5 *versus* 1	1,282	1,139 - 1,443	< 0,0001
4 *versus* 1	1,441	1,269 - 1,636	< 0,0001
2 *versus* 1	1,300	1,075 - 1,572	0,0068
Narcotics	0.838	0.777 - 0.903	< 0,0001
ICP	0.701	0.637 - 0.772	< 0,0001
ICU length of stay	1,035	1,031 - 1,039	< 0,0001
Pupil size	0.385	0.372 - 0.399	< 0,0001
Left pupil, moderate GCS			
Age	1,008	1,005 - 1,012	< 0,0001
Sex	1,181	1,047 - 1,334	0,0070
Race			
4 *versus* 1	0.531	0.407 - 0.692	< 0,0001
3 *versus* 1	0.638	0.496 - 0.821	0,0005
2 *versus* 1	1,270	1,057 - 1,526	0,0109
Primary diagnosis			
5 *versus* 1	0.582	0.485 - 0.699	< 0,0001
4 *versus* 1	0.476	0.400 - 0.567	< 0,0001
3 *versus* 1	0.596	0.447 - 0.795	0,0004
2 *versus* 1	2,141	1,292 - 3,547	0,0031
Pupil size	0.409	0.387 - 0.432	< 0,0001
Left pupil, severe GCS			
Age	0.995	0.993 - 0.998	< 0.0001
Sex	1,243	1,164 - 1,327	< 0.0001
Race			
4 *versus* 1	0.877	0.778 - 0.988	0.0305
3 *versus* 1	0.440	0.383 - 0.506	< 0.0001
2 *versus* 1	0.801	0.733 - 0.875	< 0.0001
Primary diagnosis			
5 *versus* 1	1,162	1,064 - 1,269	0.0009
3 *versus* 1	1,361	1,135 - 1,632	0.0009
2 *versus* 1	1,857	1,634 - 2,110	< 0.0001
Narcotics	0.875	0.820 - 0.934	< 0.0001
ICP	0.780	0.719 - 0.847	< 0.0001
ICU length of stay	1,009	1,007 - 1,011	< 0.0001
Pupil size	0.420	0.490 - 0.432	< 0.0001

OR - odds ratio; 95%CI - 95% confidence interval; GCS - Glasgow Coma Scale score; ICP - intracranial pressure; ICU - intensive care unit.

**Table 2B:** Statistically significant parameter estimate based on acceptable threshold dilation velocity for the right pupil

	Adjusted model		
	OR	95%CI	p value
Right pupil, mild GCS			
Age	1,016	1,013 - 1,018	< 0.0001
Sex	1,096	1,009 - 1,190	0.0299
Race			
4 versus 1	0.458	0.383 - 0.547	< 0.0001
2 versus 1	0.618	0.549 - 0.696	< 0.0001
Primary diagnosis			
5 versus 1	1,556	1,377 - 1,760	< 0.0001
4 versus 1	1,358	1,196 - 1,541	< 0.0001
3 versus 1	1,201	1,068 - 1,351	0.0022
2 versus 11	1,274	1,055 - 1,539	0.0117
Narcotics	0.868	0.804 - 0.936	0.0002
ICP	0.754	0.684 - 0.832	< 0.0001
ICU length of stay	1,028	1,025 - 1,032	< 0.0001
Pupil size	0.438	0.423 - 0.453	< 0.0001
Right pupil, moderate GCS			
Age	1,017	1,013 - 1,021	< 0.0001
Sex	1,656	1.463 - 1.873	< 0.0001
Race			
4 versus 1	0.728	0.553 - 0.958	0.0236
3 versus 1	0.575	0.451 - 0.733	< 0.0001
2 versus 1	1,275	1,063 - 1,529	0.0087
Primary diagnosis			
4 versus 1	0.573	0.483-0.678	< 0.0001
3 versus 1	0.656	0.492 - 0.876	0.0043
2 versus 1	2,256	1,366 - 3,726	0.0015
Pupil size	0.452	0.428 - 0.478	< 0.0001
Right pupil, severe GCS			
Age	0.996	0.994 - 0.998	0.0007
Sex	1,271	1,191 - 1,357	< 0.0001
Race			
4 versus 1	0.882	0.732 - 0.992	0.0009
3 versus 1	0.506	0.440 - 0.582	< 0.0001
Primary diagnosis			
5 versus 1	1,348	1,234 - 1,473	< 0.0001
3 versus 1	1,513	1,261 - 1,815	< 0.0001
2 versus 1	1,459	1,293 - 1,647	< 0.0001
Narcotics	0.876	0.821 - 0.935	< 0.0001
ICP	0.856	0.790 - 0.928	0.0002
ICU length of stay	1,010	1,007 - 1,012	< 0.0001
Pupil size	0.431	0.419 - 0.443	< 0.0001

OR - odds ratio; 95%CI - 95% confidence interval; GCS - Glasgow Coma Scale score; ICP - intracranial pressure; ICU - intensive care unit.

For severe GCS, in the left eye, all variables except diagnosis with TBI were statistically significant. In the right eye, all variables except diagnosis with TBI and being Asian were statistically significant.

These tables also present the odds ratios (OR), comparing the association between an exposure and outcome, and the confidence intervals (CIs). An OR > 1 shows a positive association (exposure leads to result), OR < 1 shows a negative association (exposure decreases the likelihood), and OR = 1 shows no influence of the exposure on the result.^(21)^

Table 3 shows the percent concordance and discordance of the ROCs with the DVs for the left and right pupils.

**Table 3:** Concordance and discordance based upon pupil and Glasgow Coma Scale

Eye examined	Adjusted model		
	% concordant (AUC)	% discordant (FPR)	Pupillary DV
Left pupil, mild GCS	83.8	16.2	1.2mm/s
Right pupil, mild GCS	80.8	19.2	
Left pupil, moderate GCS	81.2	18.8	1.1mm/s
Right pupil, moderate GCS	80.6	19.4	
Left pupil, severe GCS	80.2	19.8	0.9mm/s
Right pupil, severe GCS	79.4	20.6	

AUC - area under the curve; FPR - false positive rate; DV - dilation velocity; GCS - Glasgow Coma Scale score.

**Figure 1:**
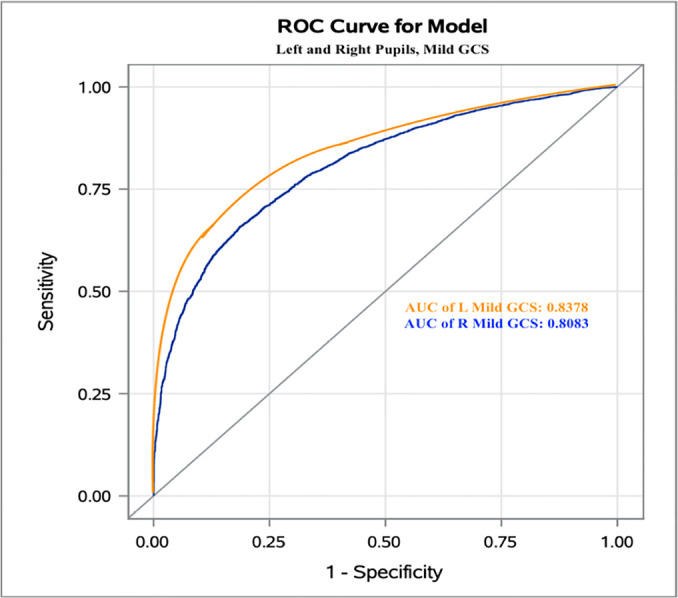
The Receiver Operating Characteristic curve of mild Glasgow Coma Scale patients and the left and right eyes. ROC - Receiver Operating Characteristic; GCS - Glasgow Coma Scale; AUC - area under the curve; L - left; R - right.

An AUC > 0.75 is considered to be statistically acceptable. Classification based on the AUC showed that for mild GCS, the DV threshold value was 1.2mm/s, with false probability rates of 0.1602 and 0.1902 and areas under the curve of 0.8380 and 0.8080 in the left and right eyes, respectively (Figure 1).

**Figure 2:**
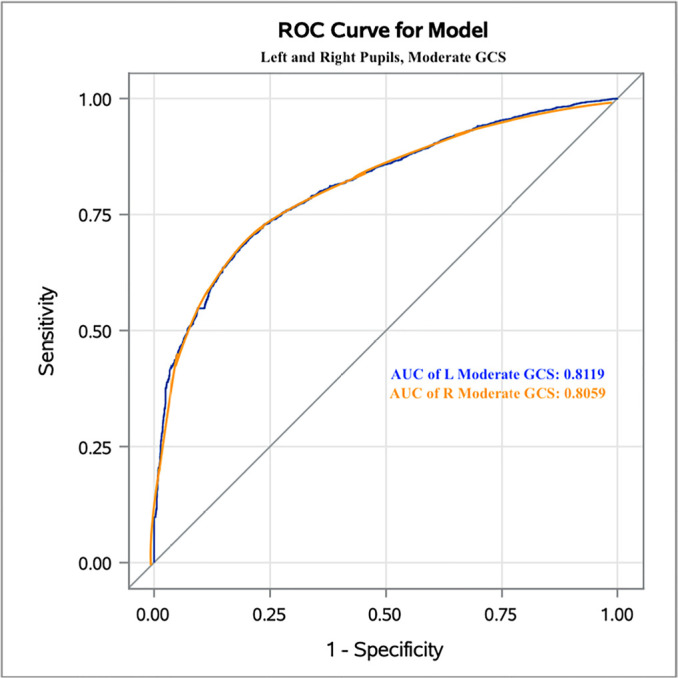
The Receiver Operating Characteristic curve for moderate Glasgow Coma Scale patients and left and right eyes. ROC - Receiver Operating Characteristic; GCS - Glasgow Coma Scale; AUC - area under the curve; L - left; R - right.

For moderate GCS, the DV threshold value was 1.1mm/s, with false probability rates of 0.188 and 0.194 and areas under the curve of 0.812 and 0.806 in the left and right eyes, respectively (Figure 2).

**Figure 3:**
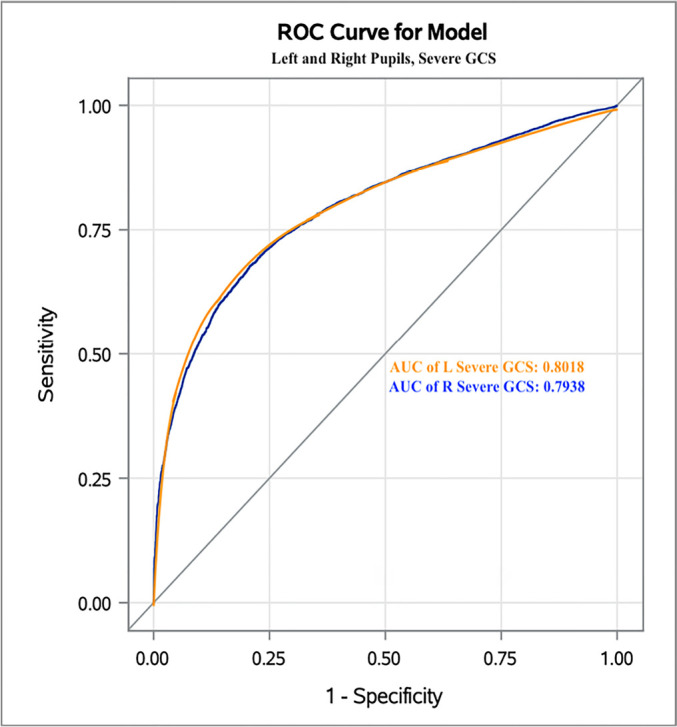
The Receiver Operating Characteristic curve for severe Glasgow Coma Scale patients and left and right eyes. ROC - Receiver Operating Characteristic; GCS - Glasgow Coma Scale; AUC - area under the curve; L - left; R - right.

For severe GCS, the DV was 0.9mm/s, with false probability rates of 0.1980 and 0.2060 and AUC of 0.8020 and 0.7940 in the left and right eyes, respectively (Figure 3). These results can be further compared to those for mild and moderate GCS (Figures 1 and 2). Furthermore, sensitivity analyses were performed to ascertain the discriminative power of the classification algorithm. A total of 66.67% of ENDPANIC registry participants were assigned to the derivation data. The remaining 33.33% were assigned to the validation. The results across the two populations resulted in parameter estimates < 10% difference.^(22)^ Similar differences were also found when comparing these estimates with those of the entire population.

## DISCUSSION

The results support the hypothesis that dilation velocity varies with the severity of brain injury.^(23^-^27)^ These findings extend those of many recent publications that have focused primarily on the NPi, a derived summary score indexed to normal. Lussier et al.^(28)^ recently published that normal DV in critically ill patients ranges significantly (0.3 - 1.1); however, this was a population average and did not utilize machine learning to assess the statistical validity based on groupings. Bergamin et al.^(29)^ found a significant difference in the response of healthy versus diseased eyes, showing that DV could indicate an underlying disease process. In this study, we decided to divide our data into three GCS classifications due to an unclear determination of DV for each range. Our finding that DV varies by GCS provides convergent validity for the studies of AIP and ICP, given that ICP also varies by GCS category.^(30, 31)^ Furthermore, this finding allows patients with a variety of GCS scores to be assessed for clinical severity through their DV.

The problem has been that there was no standardized way to assess pupillary dilation: subjective descriptions can enhance, not replace, objective data. Utilizing AIP provides discrete measures of DV and provides clinicians with a novel biomarker by which to assess certain neurological insults that place the patient at risk for secondary brain injury.^(4)^ Abnormal dilation of one or both eyes can be clinically significant and show damage or interference with nerves and related structures. Obtaining numerical measurements for pupil DVs and comparing them to a standardized critical value to determine a patient’s clinical status is crucial. Gathering the data using a pupilometer is not enough; the utilization of machine learning to calculate the AUC, ORs, and logistic regressions of the data provides us with the tools to assess the validity of DVs and their indications of clinical status. GCS confirms whether the patient has neurological impairment, and comparison with the normal DV value in that category can indicate mild to severe brain injury status. This integration of machine learning with clinical knowledge is promising for appropriate assessment and patient monitoring.^(32)^ Hence, categorization of brain injury based upon GCS makes logical sense as well. This quantitative pupillometry allows for reliable results that can be evaluated and reused in future studies.

As acknowledged by Shoyombo et al.,^(15)^ a multitude of variables are involved in the PLR. Determining prognosis from subjective analysis is inadequate. The DV itself is also inadequate but provides a foundation for further patient observation. In a study conducted by Olson et al.,^(33)^ it was shown that if there was a human disagreement between a pupil’s reactivity, when the PLR was most compromised (most abnormal), there was only 49% agreement between clinicians. This supports the idea that human observation has its own limitations; objective facts provide necessary clarity. There are numerous factors influencing DV, including the confounding factors controlled for in this study. Between each eye and GCS rating, there were different confounders that were statistically significant or not. This shows that the trends of the variables need to be monitored and considered, since various initial conditions can not only positively or negatively influence the PLR but can also affect the patient’s clinical status.

The patient’s medical diagnosis is an example of a controlled confounder. Depending on the patient’s diagnosis, various ocular neurological structures could be severely affected, drastically altering the way that the eyes process and respond to stimuli. There were five categories of diagnoses included in our analysis, each with different ORs. In this study, ORs indicated whether a specific diagnosis is associated with an abnormal DV change.^(21)^ When the OR is > 1, there is a positive association between diagnosis and influence on DV, and the converse is true of ORs < 1.

**Figure 4:**
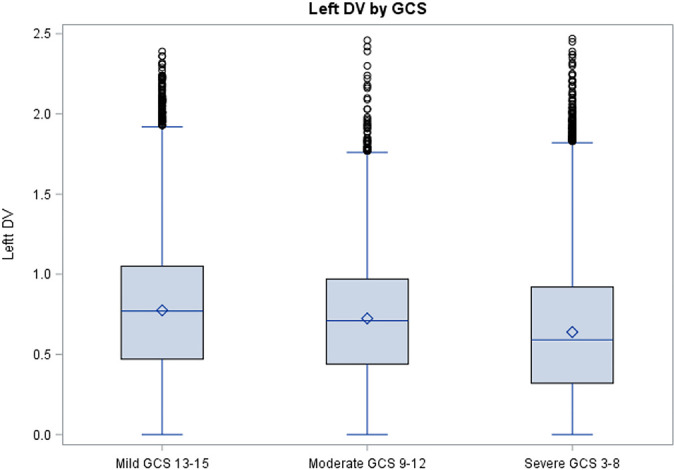
Boxplot distribution of left pupil dilation velocities based on the Glasgow Coma Scale classification. DV - dilation velocity; GCS - Glasgow Coma Scale.

**Figura 5:**
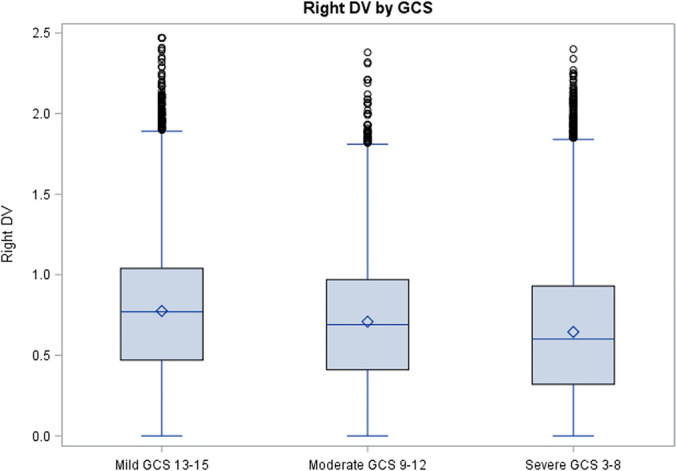
Distribuição das velocidades de dilatação pupilar com base na classificação por velocidade de dilatação pupilar. VD - velocidade de dilatação; ECG - escala de coma de Glasgow.

Regardless of how different confounding factors influence DVs, it seems evident that DVs are a marker of neuronal injury. Deviations from normal standardized values could indicate the severity of the clinical situation or even the specific condition affecting the patient. In this study, the DV calculated showed a critical cutoff of DVs above and below that value (Figures 4 and 5).

The significance of having a DV below the threshold value could indicate worsening clinical outcome. Distinguishing normal DVs from abnormal DVs can help clinicians to quickly develop diagnosis and treatment plans, thus decreasing patient mortality.

There are some noted limitations that include grouping DVs by GCS trichotomized as mild, moderate, or severe, which may result in misclassification of injury. Additionally, the sample includes primary diagnosis classifications as broad categories that include patients’ primary lesions outside of the central nervous system. However, this represents pragmatic sampling in that all patients in the sample were those who had a determined need for GCS scores (e.g., an injury likely to result in altered level of consciousness). Future studies can consider studying specific DVs based on individual diseases or can study patients based on their eye GCS value instead of their total GCS to minimize these limitations.

## CONCLUSION

As shown with the data gathered, as the Glasgow coma scale increases in severity, the dilation velocity correspondingly decreases in magnitude. The corresponding changes in the dilation velocity with certain disease processes as well as varied levels of consciousness indicate that abnormal dilation velocities are potential biomarkers of neuronal injury and potential prognosticators for the severity of presentation.

Reporting dilation velocities provides insight into patients with brain injury who are at risk for neurologic deterioration. Dilation velocity may be able to assist in determining diagnosis, prognosis, and treatments, especially when further combined with other variables used in calculating the pupillary light reflex. Future studies should focus on individual diseases and individual eye Glasgow coma scale scores, as well as differentiating the severity of injury depending on the dilation velocity classification cutoff. A better understanding of pupillary dilation velocity can further classify neuronal injury and lead to newer, more conservative ways to assess neuronal injury.
